# Clinical Studies of the Early Stages of Inebriety

**Published:** 1885-05

**Authors:** T. D. Crothers

**Affiliations:** Superintendent Walnut Lodge, Hartford, Conn


### Clinical Studies of the Early Stages of Inebriety.

By T. D. Crothers, M. D.

Superintendent Walnut Lodge, Hartford, Conn

In examining any phase of inebriety it should be remembered that we are dealing with an exceedingly complex and variable neurosis, less marked to ordinary observation than almost any other form of insanity, except in the later stages. The early symptoms are prominently masked, and seldom recognized unless the patient is under frequent observation. When the disorder has existed for years and becomes chronic, the presence of the disease is not disputed, but of the early stages, the crudest theories of science and theology are advanced in explanation. We present the following as an answer to many inquiries in relation to a premonitory stage or period preceding inebriety. Is there a continuous chain of symptoms or pathological connection, from the time of the first alcoholic poisoning to the advent of marked inebriety, that may be grouped and studied?

In examining the early causes of inebriety, we are often surprised to find how vividly the first intoxication, or conditions of alcoholic poisoning, is impressed on the memory of the patient. When every other occurrence is vague or confused, the impressions received at this time are clear. This is a significant hint of a pathological condition or change in the system at this time, influencing all the future. Our inquiries, based on a number of cases, seem to indicate three groups, which are more or less distinct in the early periods. The first one is marked by a long premonitory stage, in which, comparatively harmless drinks are used, such as sodas, cider, small beer, etc., either preceding the first intoxication or following for months or years before inebriety comes on. The second group have no special preliminary symptoms, either after the shock of intoxication or before it. Frequently they are rigid abstainers, and almost fanatical temperance men, but when they begin to drink go down precipitately.

A third group seem always on the verge of inebriety, using beer and alcohol constantly. In their conversation and manner they usually indicate a disturbed mental and physical condition.

Generally the progress of the case is very slow, and always attended with doubtful and uncertain symptoms.

The first narcotism, coma, or intoxication following the use of alcohol, seems to be the starting point for various lesions, and undoubtedly acts as a shock, profoundly impressing the organism. In some cases the march of the disorder from this period is without interruption, and readily traced, in others it is obscure, with long intervals and diversions, and in a certain number of cases the first complete intoxication is followed by a condition of repulsion, both physical and mental, and total abstinence ever after.

The term inebriety is usually applied when alcohol is used to excess continuously, or in paroxysms, without the control of the patient, or apparently so.

The following cases are presented as illustrating some of these groups:

*Case* I.—*Inebriety from Nutrient Excess in Childhood.* A. B., a lawyer, born of healthy and very wealthy parents; through childhood he was indulged in every luxury of the table that money could furnish. At sixteen he suffered from acute dyspepsia, from which recovery was very slow. During college life he was under constant medical treatment for this affection. At twenty-four he was junior partner in a large law firm, and worked very hard.

He ate carefully, used cider and various stomach remedies at this time, was ambitious and lived temperately. Two years later he married and began to drink wine at dinner. The dyspepsia passed off, and he commenced to live luxuriously; wine, beer and various bitters were used regularly, and only one hearty meal a day was taken; all other times of eating were irregular.

During the next two years he grew stout and became more fond of good dinners, and was called an epicure. On one occasion of great excitement he drank to intoxication and helplessness at the house of a friend; a low, nervous fever followed for several days, depending in part on grief at his situation, and the shock to his system. The next six months he drank nothing but cold water, and manifested great disgust for alcohol. During this time his disposition suddenly began to change; he would become abstracted in his manner in the midst of company, change the subject abruptly, manifest rudeness and neglect, or go away by himself and give no explanation. Then he began to drink tea and coffee, especially after any exertion or fatigue. A year later he entertained a delusion of some stomach disorder that no physician could understand, and sought strange remedies from strange sources, patronizing quacks and consulting fortune-tellers, etc.

The changes of disposition were more pronounced, and his manner became impulsive and jerking. He began to drink wine at dinner, and occasionally very hard at night. His temper varied often from one extreme to the other. He advocated beer selling, and made strong speeches advocating its use; at this time he professed to be a temperance man, denouncing all drinking other than ale or wine. At times he was excessively emotional, especially at home with his family. Being elected judge, he seemed to improve and have more control over his temper and emotions. A few months later, after much excitement, he drank to intoxication in his room, giving no explanation or reason, and never referring to it afterward. Often he would come home nervous and excitable, and impulsively drink tea, coffee, etc., and sometimes wine, walking the room for hours before retiring. The next day he would appear on the bench, and be perfectly self-possessed; occasionally he would answer a lawyer sharply; or in charging the jury, after defining the law with great clearness, give way to a burst of sympathy that was noticeable.

Usually he drank nothing until evening, sometimes eating very little for a day or more, then indulging in a sumptuous dinner with wines, etc. He complained of wandering pains and exhaustion at times and took much medicine, usually strong tonics. All unexpectedly he manifested a great ambition to become a member of the legislature, and resigned his judgeship and went over the district electioneering. Failing, he drank to excess for a week or more, then signed the pledge and began to lecture on temperance. From this time all his habits became more irregular, and weeks were spent in hunting and fishing; wine and whisky were used regularly, but rarely to intoxication. His mind and memory failed, and he became unreliable in social and business relations. Six years from the time of his first intoxication he was a periodical inebriate.

*Note.*—The rich diet of early life produced a train of disorders, first manifest in dyspepsia, then in perverted nutrient desires. From the time of the first intoxication there was a continuous chain of symptoms indicating progressive degeneration of both the mind and body.

*Case 2.—Inebriety from excesses following sudden wealth and changed circumstances.* C. D. No history of parents, a hard-working manufacturer, in good health, living temperately. By a fortunate patent he soon amassed a large property. The excitement and irregularities of his new situation developed a taste for wine and porter. He became profoundly intoxicated, then signed the pledge, but drank soda, lager beer and cider. All at once his character and manner changed; up to this time he was a credulous, generous man, of mild, easy disposition, and careful of his reputation, then he became suspicious and exacting, sometimes very generous, then extremely parsimonious, also impulsive, unreasonable, and involved in business quarrels with all his associates. He drank whisky at meals regularly, was reckless of his reputation, bought and drove fast horses, and frequented the society of fast men, and was an inebriate apparently without any control of himself. His wife was injured and died. On her death bed he promised to stop drinking. For one year he has continued sober, although his manner and character remain the same. This case is noted for the prominence of its symptoms, and the sudden cessation from drinking caused by the presence of profound grief. In all probability they will appear again. This patient attributes all his drinking to the adulterated wine that produced the first intoxication.

The following are examples of the second group:

*Case* 3.—*Inebriety from some obscure cause, beginning with mental shock and grief, etc.* E. F. A literary man and author, no clear history of parents, probably healthy, was a temperate man, living frugally and working hard. Having written a popular book, which made him famous, he occupied a very enviable position in the world. In the height of his prosperity his wife and child died. After the funeral he retired to his room, and for weeks refused to be seen by any one, then suddenly he drank to intoxication. From this time he began to write short stories on insanity, painting the most harrowing pictures of persons who were conscious of darkened reason, etc. Then, after drinking very hard for a time, he became a temperance man. Later he wrote another book which attracted no attention, and for two years afterward found employment gathering items for a daily paper. Nothing prominent was noticed except an occasional outburst of temperance enthusiasm or paroxysm of drinking, which ended only when his money was gone, followed by another period of sobriety, and so for two years, when he died from pneumonia. In this case many of the symptoms were obscure and unnoticed. In some of his writings there are traces of morbid impulses and delusions.

*Case* 4.—*Inebriety from Inheritance.* G. H., a commercial traveler in good health, and temperate. His father was a moderate drinker, and his mother was hysterical; he was advised by a physician to take brandy for some obscure trouble. Six months after, he became intoxicated, and so violent as to be locked up. After this he became a strong temperance man, and leader in different lodges. Nothing was noticed about him unusual except a morbid desire to indulge in all games of chance, playing cards and buying lottery tickets, being prominent at raffles, etc. Three years later he began to drink to excess, and suddenly became a furious inebriate, drinking at all times and places. In this case all the symptoms were not prominent until at last they burst out in uncontrollable drunkenness.

In the third group inebriety seems present at all times, yet there is an absence of many symptoms, and a general uncertainty of progress.

*Case* 5.—*Inebriety from bad surroundings.* F. J., a farmer, of healthy parents, a vigorous, temperate man. Went into the hotel business, sold liquor, and was avaricious to make money. After a time he became very much intoxicated, then drank daily for years, always exhaling a strong odor of alcohol, with face and eyes congested, and step unsteady; at night his voice was hoarse. His mind was clear and capable of conducting business, and his health remained about the same. He claimed to be an example of a moderate drinker who could stop at a certain limit, or having his body well under subjection, etc., attending church, and appearing to be a good citizen and Christian man. He was an ardent admirer of horse races, and spent much money and time supporting them. A few years later he became reckless of his reputation and manners, and exhibited the presence of many delusions, then drank very hard for months before death.

*Case* 6.—*Inebriety from exposure and overwork.* H. L., a physician, with insanity on his mother's side; healthy, well educated, and a strong temperate man, settled in a rough country district, and boarded at a country hotel. Was very ambitious and worked hard, driving night and day, neglecting all healthy habits of living. Began to use alcohol to keep up when business was pressing, and during a season of much excitement became helplessly intoxicated. On recovering he signed the pledge and continued sober for months, then began to drink again, but this time with great regularity, carrying a bottle in his pocket and using it at intervals, reasoning that it supplied him with lost energy, which his system demanded. He became careless in his appearance, and excessively jealous of his neighbors in the profession. His mind lost its former poise, and he either was strangely dogmatic or childishly credulous. His manners were impulsive and his disposition irritable, and the delusion of being able to stop at any time filled his mind. Four years later he became paralyzed and died.

In both of these cases the patients seemed conscious of their condition, but always asserted they were able to stop, and were never intoxicated after the first attack. They were regular drinkers and were not called inebriates in the meaning of that term. They both possessed delusions and exhibited early changes, of character and habits. To the superficial observer, their drinking had no elements of disease in it, but was more the result of vicious habits under control. Such patients always encourage this delusion of self-control, and are free to give reasons explaining their condition. Often there is a strange indifference to their condition, which is significant of profound disorders. All these cases were under the care of various physicians at different times, and were treated for rheumatism, dyspepsia, diseases of the heart, etc. All reference to the real cause, alcohol, was dismissed with the simple advice to abstain. In this way such cases are not observed until they have passed into a chronic form. The changes of the faculties are also not noticed, because the intellect seems to be as acute as ever. Only a close observation reveals the blunting of the moral nature of the man, his truthfulness particularly being changed to unblushing prevarication, and often his modesty turns into great presumption. An inclination to lead a fast life, to indulge in games of chance, a credulity that is unusual before, are important hints that cannot be mistaken. The friends and physicians who have charge of such cases should not be deceived, but act promptly, with a full understanding that the case is one of great uncertainty, and always more or less masked. In all these cases the memory of the first intoxication was clear, but of events after more or less obscurity prevailed. In Case 1, the first complete intoxication exploded, as it were, a long train of events, and fixed the form of future manifestation; the progress of the case from this time on was steady and marked. In Case 2, the changes following the first intoxication were sudden and marked. Also the equally abrupt termination, from some powerful impression on the nervous system. This was a check on the march of the disease, from which it will break out again with renewed energy. In Cases 3 and 4 the symptoms were more or less masked, but the progress of the disorder continued as before, ending at last abruptly. Such cases are quite common.

The other two cases represent a large class of very respectable men who seem to possess a repelling element, keeping the symptoms in abeyance. They are examples of a dangerous class who frequently betray all confidence of friends, and all unexpectedly break down in some condition of great weakness and depravity. The practical facts which these cases seem to indicate are that in all cases of inebriety a premonitory stage has existed, usually dating very prominently from the first attack, or condition of intoxication. Sometimes it precedes this event with great significance. The wise physician should always study such cases pathologically, and treat them with promptness and energy.

